# Specialized Home Care Needs and Models for Disabled Older Adults by Disability Level: A Cross‐Sectional Study

**DOI:** 10.1155/jonm/8917956

**Published:** 2025-12-04

**Authors:** Yitian Gao, Wanqiong Zhou, Lanshu Zhou

**Affiliations:** ^1^ College of Nursing, Shanghai University of Traditional Chinese Medicine, Shanghai, China, shutcm.edu.cn; ^2^ Shuguang Hospital Affiliated With Shanghai University of Traditional Chinese Medicine, Shanghai, China; ^3^ Key Laboratory of Geriatric Long-term Care, Naval Medical University, Shanghai, China, smmu.edu.cn

**Keywords:** cross-sectional, Kano model, physical disabilities, specialized home care

## Abstract

**Background:**

Home care systems face critical challenges: severe staff shortages, ill‐defined service content, and ineffective care needs communication, all contributing to suboptimal service delivery. Developing a severity‐stratified priority model for functionally impaired older adults enables precise resource allocation, improving both care quality and quality of life in this high‐risk population.

**Objective:**

To identify the specialized home care service needs of older adults with physical disabilities across varying disability levels and to propose strategies to optimize specialized home care services.

**Design:**

A cross‐sectional study.

**Setting and Participants:**

From October 2023 to January 2024, 550 older adults with physical disabilities participated in a disability assessment in Shanghai, China.

**Methods:**

Using the Kano model as the analytical framework, item attributes were determined through the maximum‐frequency method. Better–Worse analysis calculated the satisfaction and dissatisfaction indices for each service, and an importance matrix analysis was subsequently plotted to prioritize demand items.

**Results:**

A total of 534 complete datasets were obtained, with disability levels distributed as follows: complete self‐care (318 cases, 59.6%), mild disability (68, 12.7%), moderate disability (45, 8.4%), and severe disability (103, 19.3%). Better–Worse matrix analysis revealed distinct demand item distributions across disability levels. Overall, 3 items fell into the critical improvement zone, 2 in the advantage zone, and 12 in the maintenance zone. Specifically, complete self‐care participants showed 3 critical improvement, 1 advantage, and 10 maintenance items; mild disability had 3 critical improvement, 1 advantage, and 14 maintenance items; moderate disability demonstrated 6 critical improvement, 3 advantage, and 6 maintenance items; while severe disability exhibited 4 critical improvement, 6 advantage, and 8 maintenance items.

**Conclusions:**

Disabled older adults exhibit differentiated needs for specialized home care services based on disability severity. Effective care requires accurate disability identification and tailored prioritization of care programs aligned with impairment levels, ultimately enhancing service quality for this population.

**Relevance to Clinical Practice:**

This study analyzes differing specialized home care needs among older adults with different levels of disability in order to provide precise care and help reduce the burden of care.

**Patient or Public Contribution:**

Patients contributed through filling out questionnaires.

**Summary**

• Physical disabilities impose significant caregiving burdens on older adults. China currently faces dual challenges: the caregiver shortage and unclear specialized home care service needs.
• Physical disability does not equate to incapacity; 59.6% of this study demonstrate complete independence in activities of daily living.
• Needs for specialized home care services for the physically disabled elderly correlate with disability severity.
• We have identified the needs and priorities of specialized home care services for older adults with varying disability levels.

## 1. Introduction

As the aging process accelerates, individuals who acquired disabilities in their youth are transitioning into older adults with disabilities [1]. Combined with new disabilities emerging in older adults due to aging, disease, and injury, the population with physical disabilities is growing significantly, creating substantial economic and social burdens [2]. The Second National Sample Survey of Persons with Disabilities reveals that 6.34% of China’s population has disabilities, with 24.12 million (29.07%) experiencing physical disabilities [3]. The combined effects of demographic shifts and changing disease patterns will likely result in a substantial increase in both the number and growth rate of older adults with disabilities, particularly those aged 60 or older. Projections indicate the population of disabled adults aged 60+ will increase by over 13 million every five years between 2020 and 2035, reaching 127 million by 2050 [4].

Physical disability refers to varying degrees of impaired motor function with associated limitations in activities or social participation, encompassing diverse manifestations [5]. Unlike other disability types, older adults with physical disabilities frequently experience greater impacts on mobility, self‐care, daily activities, and social engagement due to functional limitations [6]. Conditions such as limb loss and paralysis restrict routine activities, while secondary injuries from falls and limited mobility represent common adverse events among this population [7]. These factors collectively increase both the need for and utilization of healthcare services among physically disabled older adults. As the country with the largest aging population globally, China faces a substantial older care burden [8]. Current statistics indicate over 40 million older adults with disabilities in China [9]. Applying the international standard of a 3:1 ratio of disabled older adults to caregivers would require at least 13 million caregivers. However, nationwide data show fewer than 500,000 service personnel across all older care institutions, with fewer than 20,000 licensed caregivers [9]. Surveys reveal that long‐term care services in current medical and eldercare institutions are primarily provided by professional nurses, full‐time doctors, and untrained nursing staff lacking formal caregiving qualifications [10].

Given the distinct functional limitations of physically disabled individuals, older adults with physical disabilities typically exhibit more complex and specific physiological/medical care needs compared with the general aging population [11]. Informal caregivers often struggle to adequately address the individualized and multifaceted requirements of this group. Consequently, delivering specialized home care services for older adults with physical disabilities represents a critical strategy for alleviating caregiving pressures and enhancing healthcare quality for this vulnerable demographic [12].

Global variations in older adults’ lifestyles and living arrangements correspond to differences in the nursing services they receive. Specialized home care refers to therapeutic nursing services delivered by nurses in patients’ homes, incorporating hospital‐level healthcare elements typically provided to inpatients [13, 14]. In Finland, home care encompasses both basic daily living assistance and specialized interventions such as neurological rehabilitation, care coordination, health behavior guidance, psychological support, nutritional management, safety protocols, and medication administration [15]. Japan emphasizes distinct professional competencies for family caregivers, including clinical skills, health monitoring, pharmaceutical knowledge, hygiene maintenance, and advanced interpersonal abilities such as adaptability and active listening [16]. In the United States, professional caregivers deliver skilled nursing care, including condition assessments, intravenous therapy, medication administration guidance, and wound management [17]. Canada’s approach involves third‐party agencies providing nonmedical personal care (e.g., bathing, dressing, meal preparation, and light housekeeping), while nurse‐led home care focuses on clinical needs such as chemotherapy supervision and wound treatment [18]. China has adopted a multidisciplinary home care model where specialized nurses perform clinical tasks (e.g., vital sign monitoring, invasive catheter management, wound care, specimen collection, and medication administration), while other professionals contribute physical therapy, occupational/speech therapy, nutritional counseling, and medical equipment support during home visits [19]. These teams may also offer referrals, care coordination, education, training, and counseling services.

However, the demand for specialized home care services for older adults with physical disabilities in China remains in the exploratory phase. Current research predominantly addresses rehabilitation and public health interventions rather than specialized care services, with limited focus on the specific needs of physically disabled individuals [20]. Furthermore, much of the existing literature on the care needs of older individuals with disabilities are outdated, and the unclear needs significantly compromise the quality and efficiency of professional home care [14, 21]. Updated surveys and studies are urgently needed to better understand the heterogeneous needs of this population. Prioritizing specialized home care services tailored to varying levels of disability severity is critical for alleviating caregiving burdens and enhancing healthcare quality for older adults with physical disabilities.

To address these gaps, we propose the following research questions:

RQ1 : Do older adults with physical disabilities mean disablement?
RQ2 : What are the priorities for specialized home care services for older adults with physical disabilities at different levels of disability?

To answer these questions, this study employs a cross‐sectional survey to (i) assess the ability to perform activities of daily living among older adults with physical disabilities and (ii) analyze needs and priority preferences across specialized home care services for those with varying functional disability levels.

## 2. Theoretical Foundations

This study explores the differentiated needs of older adults with physical disabilities across varying disability levels and individual characteristics, employing the Kano model—an analytical framework for categorizing and prioritizing user needs proposed by Noriaki Kano in 1984, now widely applied to evaluate patient needs in medical and nursing‐care services [22, 23]. The model categorizes individual service needs into six attribute types: must‐be (M), one‐dimensional (O), attractive (A), indifferent (I), reversing (R), and question quality (Q) [24]. The question quality (Q) attribute cannot be reliably evaluated because its provision or omission unpredictably affects user satisfaction. Therefore, the Q attribute is excluded from this analysis. The Kano model provides the methodological framework for developing this study’s assessment tool, aligning with the research objective to analyze participants’ needs prioritization.

**Figure 1:**
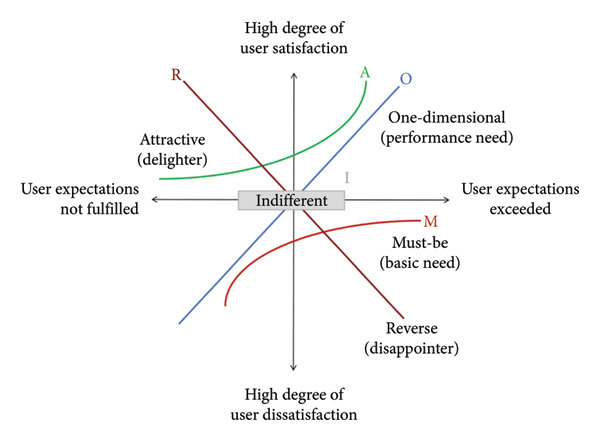
Illustration of the Kano model categories.

The five attribute categories correspond to five need classifications: M (Must‐be needs: essential services whose provision prevents dissatisfaction but does not enhance satisfaction), O (One‐dimensional needs: services where provision increases satisfaction while non‐provision maintains baseline levels), A (Attractive needs: unanticipated services that elevate satisfaction when provided without causing dissatisfaction when absent), I (Indifferent needs: services with neutral satisfaction impact regardless of provision status, requiring no prioritization), and R (Reverse needs: services whose provision reduces satisfaction while their absence improves it), as shown in Figure 1.

## 3. Methods

### 3.1. Study Design

A cross‐sectional survey was conducted from October 2023 to January 2024 among older adults with physical disabilities who underwent disability assessments in Shanghai, China. Strengthening the reporting of observational studies in epidemiology (STROBE) guidelines was followed [25].

### 3.2. Participants

#### 3.2.1. Inclusion and Exclusion Criteria

Inclusion criteria: (1) age ≥ 60 years; (2) physical disability defined by a) clinically documented motor function impairment, as confirmed by standardized disability assessments conducted by licensed physicians in Shanghai’s official evaluation system, including clinical examination, functional assessment (e.g., Modified Barthel Index), and medical history review, b) activity/participation restrictions due to structural/functional motor system damage, and c) clinical manifestations including limb paralysis, trunk paralysis, deformities, or equivalent mobility limitations; (3) ≥ 1 year of home residence with primary intention to age in place; and (4) provided voluntary written informed consent.

Exclusion criteria: (1) individuals with severe acute or life‐threatening conditions or those at high short‐term mortality risk; (2) individuals diagnosed with psychiatric or psychosomatic disorders; and (3) individuals unable to communicate effectively (e.g., due to cognitive impairment) paired with a primary caregiver lacking sufficient knowledge of their condition (e.g., temporary caregivers).

#### 3.2.2. Sample Size

Based on the Kano model requirement that distributed questionnaires must exceed 200 responses and be 5–10 times the number of questionnaire items [26], and accounting for potential errors, omissions, or invalid responses (with a 20% buffer added to the calculated sample size), the final target sample size was 232 participants. This calculation derived from 37 predefined questionnaire items.

### 3.3. Instruments

#### 3.3.1. General Information Collection Form

A sociodemographic data collection form was designed to capture participants’ baseline characteristics. The form comprised key variables including age, gender, smoking habits, alcohol use, and medical history.

#### 3.3.2. Basic Activities of Daily Living (BADL) Scale

Functional independence was assessed using the BADL scale, which evaluates six domains: bathing, dressing, toileting, indoor transferring, continence, and feeding [27]. Each item was scored as 3 (“*no, cannot perform*”), 2 (“*yes, but requires assistance*”), or 1 (“*yes, independently*”). Total scores ranged from 6 to 18, with higher scores indicating greater functional impairment. Consistent with prior studies, a BADL score ≥ 7 was defined as functional limitation. The scale demonstrated high internal consistency (Cronbach’s α = 0.85), confirming reliability.

#### 3.3.3. Kano Model–Based Needs Assessment Questionnaire for Specialized Home Care Services

The Kano model–based needs assessment questionnaire evaluated specialized home care service requirements for physically disabled older adults (Table A.1). The 37‐item instrument included domains such as psychological care, safety guidance, and clinical care needs (e.g., oral care, wound care, and intravenous fluids) [28, 29]. Each functional need was presented as paired positive (“functional form”) and negative (“dysfunctional form”) questions. Responses were scored on a 5‐point Likert scale: (1) Very satisfied, (2) Satisfied, (3) Neutral, (4) Acceptable, and (5) Not satisfied.

The questionnaire demonstrated strong reliability (Cronbach’s α > 0.7) and validity (KMO > 0.6; Bartlett’s sphericity test *p* < 0.05). Factor analysis of the 74 paired questions (37 service items × 2 forms) identified 12 factors with a cumulative variance contribution rate of 73.624%. Maximum variance rotation produced a factor loading matrix where all items exceeded 0.4 loading coefficients within their respective factors, confirming robust structural validity.

### 3.4. Data Collection and Quality Control

Considering the unique characteristics of the study population, this research employed paper‐based questionnaires for data collection. After obtaining informed consent, researchers conducted one‐on‐one interviews to assist participants in completing the questionnaires. For those unable to respond independently, primary caregivers with regular, close involvement in daily care (e.g., coresiding family members) completed the questionnaire on their behalf.

Prior to formal data collection, a pilot study with 10 participants was conducted at the target site. This yielded two key improvements: (1) survey methodology refinement through adjusted communication approaches, attitudes, and questioning techniques based on on‐site observations and (2) questionnaire optimization via modified item phrasing informed by participant feedback during pilot testing.

Three uniformly trained researchers conducted the formal survey. All three investigators received uniform training in neutral question delivery, response recording, and study protocols to minimize bias and ensure consistency. All investigators demonstrated strong communication skills, thorough familiarity with study objectives and questionnaire content, and maintained professional courtesy throughout the process to ensure efficient and accurate data collection.

### 3.5. Data Analysis

Data entry was performed using EpiData 3.1 software with dual‐entry verification. Analyses used SPSS 26.0. Normality and chi‐square tests were conducted. (1) Descriptive statistics: categorical variables were summarized as frequencies and percentages. (2) Kano attribute categorization: attributes classified per Table A.2, finalized via majority frequency. (3) Better‐Worse analysis: Satisfaction Index (SI) and Dissatisfaction Index (DI) were calculated as SI = (*A* + *O*)/(*A* + *O* + *M* + *I*), DI = (−1) (*O* + *M*)/(*A* + *O* + M + I). SI (0–1) indicates expectation intensity (higher = greater satisfaction if met); DI (−1–0) reflects dependency (lower = greater dissatisfaction if unmet). Service priorities were determined using SI (*y*‐axis) and |DI| (*x*‐axis) in a matrix with 0.5 thresholds. Quadrant I (Advantage Zone): high satisfaction gain when services are provided. Quadrant II (Maintenance Zone): services moderately improve satisfaction when provided but cause minimal dissatisfaction if omitted. Quadrant III (Secondary Improvement Zone): services have negligible impact on satisfaction regardless of provision. Quadrant IV (Critical Improvement Zone): severe dissatisfaction if service needs are unmet. Prioritization order: Quadrant IV > Quadrant I > Quadrant II > Quadrant III (Figure A.1). Items in Quadrant 4 must be provided mandatorily, while those in Quadrants 1 and 2 should be prioritized. Needs in Quadrant 3 do not require immediate prioritization.

### 3.6. Ethics

Prior to conducting the formal survey, investigators obtained informed consent from all participants, who acknowledged and consented to the use of survey data for research analysis. The original data of the study were strictly confidential and anonymized (except the patient’s name and hospitalization number), which was only used for the analysis of this study and can only be accessed by the research members of this project. This study received ethical approval from the Naval Medical University Ethics Committee (2022‐08‐16).

## 4. Results

### 4.1. Participant Characteristics

A total of 550 questionnaires were collected, with 16 excluded as invalid responses (due to incomplete information or contradictory answers to paired questions). This yielded 534 valid questionnaires, representing a 97.0% response validity rate. Among the 534 participants, 230 (43.1%) were male and 304 (56.9%) female. Disability severity levels were categorized as follows: 318 (59.6%) with complete self‐care ability, 68 (12.7%) mild disability, 45 (8.4%) moderate disability, and 103 (19.3%) severe disability. The sample included 8 distinct disability categories (Table 1).

**Table 1:** General characteristics of participants.

Variables	Categorization	*n*	%
Gender	Male	230	43.1
	Female	304	56.9
Ethnicity	Han	527	98.7
	Manchu	5	0.9
	Tujia	1	0.2
	Shui	1	0.2
Education	Below junior high school	307	57.5
	High school/junior college	93	17.4
	University college	61	11.4
	Undergraduate	69	12.9
	Graduate student and above	4	0.7
Specific site of loss of limb dysfunction	Left upper limb	174	32.6
	Left lower limb	179	33.5
	Right upper limb	75	14
	Right lower limb	183	34.3
	Paraplegia	12	2.2
	Left hemiplegia	32	6
	Right hemiplegia	26	4.9
	Total paralysis	12	2.2
Type of co‐occurring disability	Speech disability	67	12.5
	Mental disability	28	5.2
	Hearing disability	40	7.5
	Intellectual disability	16	3
	Visual disability	30	5.6
	Physical disability	138	25.8
ADL rating	Fully self‐care	318	59.6
	Mildly disability	68	12.7
	Moderate disability	45	8.4
	Severe disability	103	19.3

*Note*: *n*: frequencies; %: percentages.

### 4.2. Kano Attribute Analysis of Specialized Home Care Services Across Disability Levels

The 37 specialized home care service items were categorized according to their Kano attributes using the Kano assessment framework (Table A.2). Kano attributes were calculated for each participant’s service needs. The frequency of each Kano attribute per service item was analyzed, with the most frequent attribute identified as the definitive classification for that item.

#### 4.2.1. Specialized Home Care Services Needs for all Older Adults With Physical Disabilities in This Study

On the whole, the analysis identified four items as must‐be needs (psychological care, functional rehabilitation training guidance, healthcare knowledge guidance, and sleep nursing), one item as one‐dimensional needs (prevention and management of falls), nine items classified as attractive needs (nursing knowledge and skills guidance, safety guidance, medication guidance, guidance on the use and selection of instruments, disease prevention knowledge guidance, safety risk assessment for older adults, nutritional support nursing, assisted defecation, and acupoint massage), and 21 items as indifferent needs (Table A.3).

#### 4.2.2. Specialized Home Care Services Needs for Fully Self‐Care Older Adults With Physical Disabilities

Among the service needs of fully self‐care older adults with physical disabilities, analysis identified three must‐be needs (psychological care, healthcare knowledge guidance, and sleep nursing), one item as one‐dimensional needs (prevention and management of falls), nine attractive needs (nursing knowledge and skills guidance, safety guidance, medication guidance, guidance on the use and selection of instruments, disease prevention knowledge guidance, safety risk assessment for older adults, nutritional support nursing, assisted defecation, and acupoint massage), and 24 indifferent needs (Table A.4).

#### 4.2.3. Specialized Home Care Services Needs for Mildly Disabled Older Adults With Physical Disabilities

Among the service needs of mildly disabled older adults with physical disabilities, four must‐be needs (psychological care, functional rehabilitation training guidance, healthcare knowledge guidance, and sleep nursing), two one‐dimensional needs (prevention and management of falls, and maintenance and care of living aids), ten attractive needs (nursing knowledge and skills guidance, safety guidance, medication guidance, guidance on the use and selection of instruments, disease prevention knowledge guidance, safety risk assessment for older adults, nutritional support nursing, assisted defecation, rehabilitation training, and acupoint massage), and seventeen indifferent needs were identified (Table A.5).

#### 4.2.4. Specialized Home Care Services Needs for Moderately Disabled Older Adults With Physical Disabilities

Among the service needs of moderately disabled older adults with physical disabilities, six must‐be needs (psychological care, functional rehabilitation training guidance, sleep nursing, prevention and management of pressure injuries, prevention and management of falls, and rehabilitation training), two one‐dimensional needs (maintenance and care of living aids and supervision of medical behavior), seven attractive needs (nursing knowledge and skills guidance, safety guidance, disease prevention knowledge guidance, healthcare knowledge guidance, safety risk assessment for older adults, oral care, and acupoint massage), and twenty two indifferent needs were categorized (Table A.6).

#### 4.2.5. Specialized Home Care Services Needs for Severely Disabled Older Adults With Physical Disabilities

Among the service needs of severely disabled older adults with physical disabilities, six must‐be needs (psychological care, sleep nursing, catheterization nursing, prevention and management of pressure injuries, prevention and management of falls, and rehabilitation training), four one‐dimensional needs (functional rehabilitation training guidance, oral care, maintenance and care of living aids, and supervision of medical behavior), six attractive needs (nursing knowledge and skills guidance, safety guidance, disease prevention knowledge guidance, healthcare knowledge guidance, safety risk assessment for older adults, and acupoint massage), and twenty one indifferent needs were observed (Table A.7).

### 4.3. Better–Worse Analysis

#### 4.3.1. Better–Worse Coefficient

SI and DI for different specialized home care service items were calculated according to Better–Worse. Results indicate that among all older adults with physical disabilities in this study, SI for service needs ranges from 0.247 to 0.905 and DI ranges from −0.594 to −0.091; among the fully self‐care older adults with physical disabilities, SI ranges from 0.241 to 0.908 and DI ranges from −0.726 to −0.071; among the mildly disabled older adults with physical disabilities, SI ranges from 0.200 to 0.953 and DI ranges from −0.791 to −0.0154; among the moderately disabled older adults with physical disabilities, SI ranges from 0.233 to 0.884 and DI ranges from −0.791 to −0.070; and among the severely disabled older adults with physical disabilities, SI ranges from 0.257 to 0.932 and DI ranges from −0.737 to −0.165 (Table 2).

**Table 2:** Satisfaction and dissatisfaction index of specialized home care service items for older adults with physical disabilities.

Items	All		Fully self‐care		Mildly disability		Moderate disability		Severe disability	
	SI	DI	SI	DI	SI	DI	SI	DI	SI	DI
1.	0.282	−0.581	0.260	−0.520	0.305	−0.644	0.250	−0.682	0.347	−0.683
2.	0.774	−0.178	0.819	−0.148	0.750	−0.132	0.682	−0.227	0.693	−0.277
3.	0.821	−0.333	0.849	−0.311	0.838	−0.250	0.682	−0.364	0.784	−0.451
4.	0.398	−0.564	0.334	−0.463	0.358	−0.791	0.372	−0.791	0.628	−0.628
5.	0.715	−0.267	0.815	−0.297	0.758	−0.333	0.442	−0.140	0.505	−0.188
6.	0.6	−0.109	0.651	−0.091	0.621	−0.061	0.429	−0.143	0.500	−0.184
7.	0.88	−0.304	0.891	−0.273	0.953	−0.266	0.837	−0.442	0.816	−0.367
8.	0.59	−0.594	0.495	−0.726	0.500	−0.727	0.591	−0.477	0.932	−0.165
9.	0.284	−0.096	0.279	−0.074	0.246	−0.046	0.318	−0.136	0.307	−0.178
10.	0.303	−0.104	0.328	−0.093	0.200	−0.015	0.279	−0.116	0.303	−0.192
11.	0.322	−0.098	0.343	−0.083	0.254	−0.016	0.318	−0.091	0.303	−0.202
12.	0.905	−0.262	0.908	−0.242	0.937	−0.238	0.884	−0.302	0.885	−0.323
13.	0.442	−0.295	0.322	−0.184	0.407	−0.153	0.619	−0.524	0.735	−0.602
14.	0.274	−0.118	0.275	−0.100	0.231	−0.077	0.302	−0.140	0.286	−0.194
15.	0.267	−0.104	0.273	−0.088	0.246	−0.046	0.279	−0.140	0.257	−0.178
16.	0.285	−0.116	0.287	−0.095	0.308	−0.077	0.273	−0.159	0.270	−0.190
17.	0.421	−0.158	0.462	−0.147	0.425	−0.050	0.333	−0.139	0.360	−0.253
18.	0.283	−0.114	0.280	−0.100	0.317	−0.050	0.286	−0.119	0.267	−0.188
19.	0.592	−0.091	0.651	−0.071	0.603	−0.032	0.386	−0.091	0.505	−0.190
20.	0.263	−0.092	0.262	−0.074	0.266	−0.063	0.233	−0.070	0.278	−0.175
21.	0.248	−0.537	0.241	−0.505	0.227	−0.576	0.273	−0.500	0.270	−0.630
22.	0.297	−0.198	0.272	−0.101	0.292	−0.092	0.372	−0.116	0.343	−0.596
23.	0.363	−0.21	0.376	−0.234	0.410	−0.077	0.297	−0.108	0.338	−0.270
24.	0.346	−0.331	0.405	−0.179	0.302	−0.032	0.256	−0.535	0.300	−0.720
25.	0.389	−0.151	0.449	−0.142	0.390	−0.098	0.290	−0.105	0.299	−0.221
26.	0.599	−0.176	0.662	−0.171	0.625	−0.109	0.390	−0.146	0.475	−0.248
27.	0.25	−0.097	0.245	−0.074	0.239	−0.075	0.279	−0.093	0.258	−0.186
28.	0.617	−0.556	0.668	−0.521	0.646	−0.385	0.419	−0.628	0.515	−0.737
29.	0.322	−0.177	0.283	−0.217	0.298	−0.053	0.351	−0.108	0.432	−0.168
30.	0.288	−0.158	0.284	−0.148	0.258	−0.106	0.317	−0.171	0.316	−0.237
31.	0.516	−0.289	0.541	−0.287	0.561	−0.273	0.302	−0.140	0.500	−0.370
32.	0.401	−0.19	0.420	−0.165	0.415	−0.146	0.324	−0.162	0.383	−0.284
33.	0.41	−0.297	0.359	−0.139	0.546	−0.455	0.326	−0.558	0.505	−0.545
34.	0.61	−0.264	0.583	−0.293	0.567	−0.224	0.628	−0.163	0.719	−0.240
35.	0.337	−0.207	0.351	−0.236	0.313	−0.119	0.341	−0.091	0.304	−0.226
36.	0.564	−0.325	0.473	−0.204	0.652	−0.394	0.811	−0.649	0.692	−0.539
37.	0.54	−0.304	0.443	−0.151	0.682	−0.424	0.614	−0.500	0.728	−0.630

Abbreviations: SI, satisfaction index; DI, dissatisfaction index.

#### 4.3.2. Prioritization of Specialized Home Care Services Needs Based on Better–Worse Matrix

##### 4.3.2.1. Specialized Home Care Services Needs for all Older Adults With Physical Disabilities in This Study

Overall, the distribution of home care service needs for older adults with physical disabilities was as follows: critical improvement zone (3 items): psychological care, functional rehabilitation training guidance, and sleep nursing; advantage zone (2 items): healthcare knowledge guidance and prevention and management of falls; maintenance zone (12 items); and secondary improvement zone (20 items). See Figure A.2 and Table 3 for details.

**Table 3:** Distribution of Better–Worse matrix of specialized home care service needs of older adults with physical disabilities by degree of disability.

	Quadrant 1 (advantage zone)	Quadrant 2 (maintenance zone)	Quadrant 3 (secondary improvement zone)	Quadrant 4 (critical improvement zone)
Overall	8, 28	2, 3, 5, 6, 7, 12, 19, 26, 31, 34, 36, 37	9, 10, 11, 13, 14, 15, 16, 17, 18, 20, 22, 23, 24, 25, 27, 29, 30, 32, 33, 35	1, 4, 21
Fully self‐care	28	2, 3, 5, 6, 7, 12, 19, 26, 31, 34	4, 9, 10, 11, 13, 14, 15, 16, 17, 18, 20, 22, 23, 24, 25, 27, 29, 30, 32, 33, 35, 36, 37	1, 8, 21
Mildly disability	8	2, 3, 5, 6, 7, 12, 19, 26, 28, 31, 33, 34, 36, 37	9, 10, 11, 13, 14, 15, 16, 17, 18, 20, 22, 23, 24, 25, 27, 29, 30, 32, 35	1, 4, 21
Moderate disability	13, 36, 37	2, 3, 7, 8, 12, 34	5, 6, 9, 10, 11, 14, 15, 16, 17, 18, 19, 20, 22, 23, 25, 26, 27, 29, 30, 31, 32, 35	1, 4, 21, 24, 28, 33
Severe disability	4, 13, 28, 33, 36, 37	2, 3, 5, 7, 8, 12, 19, 34	6, 9, 10, 11, 14, 15, 16, 17, 18, 20, 23, 25, 26, 27, 29, 30, 31, 32, 35	1, 21, 22, 24

*Note*: 1: psychological care, 2: nursing knowledge and skills guidance, 3: safety guidance, 4: functional rehabilitation training guidance, 5: medication (medication safety) guidance, 6: guidance on the use and selection of instruments, 7: disease prevention knowledge guidance, 8: health care knowledge guidance, 9: first aid knowledge and skills guidance, 10: blood glucose measurement, 11: measurement of vital signs, 12: safety risk assessment for older adults, 13: oral care, 14: wound care, 15: injectables, 16: intravenous fluids, 17: blood specimen collection, 18: perineal care, 19: nutritional support nursing, 20: physical cooling, 21: sleep nursing, 22: catheterization nursing, 23: nasogastric care, 24: prevention and management of pressure injuries, 25: ostomy and fistula care, 26: assisted defecation, 27: nebulized inhalation, 28: prevention and management of falls, 29: urination care, 30: sputum discharge care, 31: oxygen therapy, 32: nursing care of indwelling tubes, 33: rehabilitation training, 34: acupoint massage, 35: application and cupping, 36: maintenance and care of living aids, 37: supervision of medical behavior.

##### 4.3.2.2. Specialized Home Care Services Needs for Fully Self‐Care Older Adults With Physical Disabilities

Among the fully self‐care older adults with physical disabilities, the distribution of home care service needs was as follows: critical improvement zone (3 items): psychological care, healthcare knowledge guidance, and sleep nursing; advantage zone (1 items): prevention and management of falls; maintenance zone (10 items); and secondary improvement zone (23 items) (Table 3 and Figure A.3).

##### 4.3.2.3. Specialized Home Care Services Needs for Mildly Disabled Older Adults With Physical Disabilities

Among the mildly disabled older adults with physical disabilities, the distribution of home care service needs was as follows: Critical improvement zone (3 items): psychological care, functional rehabilitation training guidance, and sleep nursing; advantage zone (1 items): healthcare knowledge guidance; maintenance zone (14 items); and secondary improvement zone (19 items) (Table 3 and Figure A.4).

##### 4.3.2.4. Specialized Home Care Services Needs for Moderately Disabled Older Adults With Physical Disabilities

Among the moderately disabled older adults with physical disabilities, the distribution of home care service needs was as follows: critical improvement zone (6 items): psychological care, functional rehabilitation training guidance, sleep nursing, prevention and management of pressure injuries, prevention and management of falls, and rehabilitation training; advantage zone (3 items): oral care, maintenance and care of living aids, and supervision of medical behavior; maintenance zone (6 items); and secondary improvement zone (22 items) (Table 3 and Figure A.5).

##### 4.3.2.5. Specialized Home Care Services Needs for Severely Disabled Older Adults With Physical Disabilities

Among the severely disabled older adults with physical disabilities, the distribution of home care service needs was as follows: critical improvement zone (4 items): psychological care, sleep nursing, catheterization nursing, and prevention and management of pressure injuries; advantage zone (6 items): functional rehabilitation training guidance, oral care, prevention and management of falls, rehabilitation training, maintenance and care of living aids, and supervision of medical behavior; maintenance zone (8 items); and secondary improvement zone (19 items) (Table 3 and Figure A.6).

### 4.4. Hierarchical Model for Prioritizing Specialized Home Care Service Needs in Older Adults With Physical Disabilities

The prioritization sequence across the four quadrants follows this order: Quadrant 4 > Quadrant 1 > Quadrant 2 > Quadrant 3. Within individual quadrants, items are ranked using the criterion: “higher SI values receive priority, with DI values determining ranking when SI values are equivalent.” This study established the specialized home care services needs hierarchy for older adults with physical disabilities by integrating matrix diagrams with SI and DI values. As Quadrant 3 items require neither prioritization nor service provision, the final hierarchical model exclusively incorporates items from Quadrants 1, 2, and 4.

#### 4.4.1. Priority Hierarchical Model for all Older Adults With Physical Disabilities in This Study

Overall, the model identifies the following top five specialized home care service priorities for older adults with physical disabilities: Item 4 (functional rehabilitation training guidance) > Item 1 (psychological care) > Item 21 (sleep nursing) > Item 8 (healthcare knowledge guidance) > Item 28 (prevention and management of falls) (Table 4).

**Table 4:** Hierarchical model for prioritizing specialized home care service needs in all older adults with physical disabilities.

Quadrant	Items	Priority ranking
Critical improvement zone	Item 4: Functional rehabilitation training guidance	1
	Item 1: Psychological care	2
	Item 21: Sleep nursing	3
Advantage zone	Item 8: Healthcare knowledge guidance	4
	Item 28: Prevention and management of falls	5
Maintenance zone	Item 12: Safety risk assessment for older adults	6
	Item 7: Disease prevention knowledge guidance	7
	Item 3: Safety guidance	8
	Item 2: Nursing knowledge and skills guidance	9
	Item 5: Medication (medication safety) guidance	10
	Item 34: Acupoint massage	11
	Item 6: Guidance on the use and selection of instruments	12
	Item 26: Assisted defecation	13
	Item 19: Nutritional support nursing	14
	Item 36: Maintenance and care of living aids	15
	Item 37: Supervision of medical behavior	16
	Item 31: Oxygen therapy	17

#### 4.4.2. Priority Hierarchical Model for Fully Self‐Care Older Adults With Physical Disabilities

Among the fully self‐care older adults with physical disabilities, the model identifies the following top five care priorities: Item 8 (healthcare knowledge guidance) > Item 1 (psychological care) > Item 21 (sleep nursing) > Item 28 (prevention and management of falls) > Item 12 (safety risk assessment for older adults) (Table A.8).

#### 4.4.3. Priority Hierarchical Model for Mildly Disability Older Adults With Physical Disabilities

Among the mildly disability older adults with physical disabilities, the model identifies the following top five care priorities: Item 4 (functional rehabilitation training guidance) > Item 1 (psychological care) > Item 21 (sleep nursing) > Item 8 (healthcare knowledge guidance) > Item 7 (disease prevention knowledge guidance) (Table A.9).

#### 4.4.4. Priority Hierarchical Model for Moderate Disability Older Adults With Physical Disabilities

Among the moderate disability older adults with physical disabilities, the model identifies the following top five care priorities: Item 28 (prevention and management of falls) > Item 4 (functional rehabilitation training guidance) > Item 33 (rehabilitation training) > Item 21 (sleep nursing) > Item 24 (prevention and management of pressure injuries) (Table A.10).

#### 4.4.5. Priority Hierarchical Model for Severe Disability Older Adults With Physical Disabilities

Among the severe disability older adults with physical disabilities, the model identifies the following top five care priorities: Item 1 (psychological care) > Item 22 (catheterization nursing) > Item 24 (prevention and management of pressure injuries) > Item 21 (sleep nursing) > Item 13 (oral care) (Table A.11).

## 5. Discussion

### 5.1. Functional Dependence Profile of Older Adults With Physical Disabilities

Among 534 older adults with physical disabilities, 216 (40.4%) exhibited functional dependence, with nearly half (47.7%) classified as severe. The rate substantially exceeds the 11.5% reported by Kuvalekar et al. in Udupi Taluk [30], indicating higher prevalence and severity in our cohort and suggesting prolonged health challenges among this population in China. In addition, aging‐related physiological decline increases vulnerability to disability progression [31], highlighting the need for proactive preventive care and nursing interventions.

As Chinese older adults with physical disabilities mainly reside at home [32], and given rapid population aging and a shortage of formal caregivers, developing specialized home‐based care programs is essential. Cost‐effective service models are critically needed, guided by patient‐centered quality metrics. This study used the Kano model—a health service prioritization framework—to assess professional home care needs through validated multidimensional assessments [33]. By categorizing services using hierarchical quality attributes, we provide evidence‐based recommendations for enhancing care‐related quality of life and optimizing community‐based services.

### 5.2. Overall Needs for Specialized Home Care Services

Notably, contrary to conventional assumptions, physical disability does not invariably translate into high demand for specialized home care services. In our study, 318 participants (59.6%) remained independent in daily living, while 12.7% had mild functional impairment. This shows that most physically disabled older adults retain self‐care autonomy despite significant disability burden. Manton et al. established that care needs correlate strongly with disability severity, which helps explain the reduced demand among more independent older adults [34]. Educational disparities further compound this trend: studies indicate that older adults with higher health literacy utilize health resources more effectively and exhibit better self‐management capabilities [35]. Our findings further underscore the importance of rational service planning and health education in addressing the nuanced needs of this population. Physical disability does not necessarily imply helplessness or passive receipt of care. Therefore, we recommend targeted health literacy programs and skill‐building workshops to enhance care engagement. Community interventions should prioritize accessible education and diversified support systems to address evolving care needs.

### 5.3. Variations in Specialized Home Care Services by Level of Functional Dependence

Severe mobility limitations increase demand for therapeutic care. Specifically, oral care, catheterization care, and rehabilitation training emerged as imperative or desired needs. Catheterization care—critical for bedridden individuals with urinary retention—was a mandatory need for those with severe functional dependence but undifferentiated for others, underscoring the necessity for caregivers to prioritize infection prevention in high‐severity cases [36]. Poor oral health, linked to frailty mechanisms, exacerbates nutritional deficits and disability risk due to impaired chewing [37].This explains why oral care was undifferentiated among independent or mildly dependent individuals but a must‐be or one‐dimensional need for those with moderate or severe dependence.

This study demonstrates that specialized home care services for older adults with physical disabilities should first assess incapacity severity and then apply the corresponding hierarchical prioritization model to determine care service allocation. Given the dual challenges of limited community health management capacity and caregiver shortages, this approach enables efficient prioritization of care needs, thereby improving service quality and old adults’ well‐being [13, 20]. For instance, medical compliance supervision was an undifferentiated need for independent or mildly dependent individuals but a one‐dimensional need for moderate‐to‐severe cases. This reflects greater caregiver dependence among those with severe disabilities, who often perceive compliance support as integral to safety [38]. Conversely, independent individuals may interpret such supervision as intrusive rather than supportive.

These insights support the development of a hierarchical prioritization model for the older adults with physical disabilities [39], where care services are systematically matched to objectively assessed disability levels. Given the constraints of limited community health capacity and caregiver shortages [40], such a model enables rational triage of services, ensuring that high‐impact interventions are directed to those with the greatest need. This approach moves beyond descriptive need assessment to offer a structured, scalable framework for precision care planning.

### 5.4. Limitations

This study assessed functional dependence through self‐reported questionnaires. While this method facilitated broader participant inclusion, potential discrepancies between self‐reported and clinically diagnosed disability status may introduce measurement bias. Additionally, the single‐country, single‐population design limits generalizability to global populations. Future multicenter longitudinal studies should dynamically monitor evolving preferences for specialized home care services across diverse populations and timeframes, informing sustainable service development.

## 6. Conclusion

This cross‐sectional study quantified needs for specialized home care services across levels of functional dependence in older adults with physical disabilities. Significant variations in care priorities were observed across dependence groups, underscoring the necessity for accurate functional assessments to guide tailored interventions. Precision in matching service provision to validated hierarchical needs may optimize care quality and outcomes in this vulnerable population.

## Ethics Statement

The study was approved by the Ethics Committee of Naval Medical University (2022‐08‐16).

## Disclosure

All authors have read and approved the final manuscript.

## Conflicts of Interest

The authors declare no conflicts of interest.

## Author Contributions

Yitian Gao: conceptualization, roles/writing–original draft, and role/writing–revising manuscripts. Wanqiong Zhou: statistical analysis and role/writing–revising manuscripts. Lanshu Zhou: supervision, funding acquisition, and writing–review and editing. Lanshu Zhou is the guarantor for this study. The corresponding author attests that all listed authors meet authorship criteria and that no others meeting the criteria have been omitted.

Yitian Gao and Wanqiong Zhou have contributed equally to this work and share first authorship.

## Funding

This study was funded by the Key Laboratory of Geriatric Long‐term Care, Naval Medical University, Ministry of Education (2023‐03), 2023‐03, and the Shanghai Key Laboratory of Health Identification and Assessment, 21DZ2271000.

## General Statement

The authors have checked to make sure that the submission conforms as applicable to the Journal’s statistical guidelines described here. The author, Wanqiong Zhou, has undergone systematic training in statistical methods and obtained a certificate of completion. She is responsible for all statistical analysis and verification work in this study.

## Supporting Information

The following are the supporting data related to this article, and all supporting information are included in Document “Supporting information”:

Supporting information Figure A.1: Better–Worse matrix analysis model (page 2 in Supporting information).

Supporting information Figure A.2: Better–Worse matrix of specialized home care service needs for all older adults with physical disabilities (page 2 in Supporting information).

Supporting information Figure A.3: Better–Worse matrix of specialized home care service needs for fully self‐care older adults with physical disabilities (page 3 in Supporting information).

Supporting information Figure A.4: Better–Worse matrix of specialized home care service needs for the mildly disabled older adults with physical disabilities (page 3 in Supporting information).

Supporting information Figure A.5: Better–Worse matrix of specialized home care service needs for the moderately disabled older adults with physical disabilities (page 4 in Supporting information).

Supporting information Figure A.6.: Better–Worse matrix of specialized home care service needs for the severely disabled older adults with physical disabilities (page 4 in Supporting information).

Supporting information Table A.1: Needs assessment questionnaire for specialized home care services (page 5–11 in Supporting information).

Supporting information Table A.2: Kano assessment framework (page 12 in Supporting information).

Supporting information Table A.3: Kano attributes of specialized home care services needs for older adults with physical disabilities (page 13 in Supporting information).

Supporting information Table A.4: Kano attributes of specialized home care services needs for fully self‐care older adults with physical disabilities (page 14 in Supporting information).

Supporting information Table A.5: Kano attributes of specialized home care services needs for mildly disabled older adults with physical disabilities (page 15 in Supporting information).

Supporting information Table A.6: Kano attributes of specialized home care services needs for moderately disabled older adults with physical disabilities (page 16 in Supporting information).

Supporting information Table A.7: Kano attributes of specialized home care services needs for severely disabled older adults with physical disabilities (page 17 in Supporting information).

Supporting information Table A.8: Hierarchical model for prioritizing specialized home care service needs in fully self‐care older adults with physical disabilities (page 18 in Supporting information).

Supporting information Table A.9: Hierarchical model for prioritizing specialized home care service needs in mildly disability older adults with physical disabilities (page 19 in Supporting information).

Supporting information Table A.10: Hierarchical model for prioritizing specialized home care service needs in moderate disability older adults with physical disabilities (page 20 in Supporting information).

Supporting information Table A.11: Hierarchical model for prioritizing specialized home care service needs in severe disability older adults with physical disabilities (page 21 in Supporting information).

## Supporting information


**Supporting Information** Additional supporting information can be found online in the Supporting Information section.

## Data Availability

The data that support the findings of this study are available from the corresponding author upon reasonable request.
