# Supplementary material for: Specialized Home Care Needs and Models for Disabled Older Adults by Disability Level: A Cross‐Sectional Study
Source: J Nurs Manag. 2025 Dec 4;2025:8917956. doi: 10.1155/jonm/8917956 (PMC12698254; doi:10.1155/jonm/8917956)
Supplement: Supplementary file 1 — Supporting Information Additional supporting information can be found online in the Supporting Information section. [file JONM-2025-8917956-s001.pdf]

Supplement: Supplementary materials to "Specialized Home Care Needs and Models for Disabled Older Adults by Disability Level: A Cross-Sectional Study"

Table of content

Figure A.1 Better-Worse matrix analysis model 2
Figure A.2 Better-Worse matrix of specialized home care service needs for all older adults with physical disabilities 2
Figure A.3 Better-Worse matrix of specialized home care service needs for fully self-care older adults with physical disabilities 3
Figure A.4 Better-Worse matrix of specialized home care service needs for the mildly disabled older adults with physical disabilities 3
Figure A.5 Better-Worse matrix of specialized home care service needs for the moderately disabled older adults with physical disabilities 4
Figure A.6. Better-Worse matrix of specialized home care service needs for the severely disabled older adults with physical disabilities 4
Table A.1 Needs assessment questionnaire for specialized home care services 5
Table A.2 Kano assessment framework 12
Table A.3 Kano attributes of specialized home care services needs for older adults with physical disabilities 13
Table A.4 Kano attributes of specialized home care services needs for fully self-care older adults with physical disabilities 14
Table A.5 Kano attributes of specialized home care services needs for mildly disabled older adults with physical disabilities 15
Table A.6 Kano attributes of specialized home care services needs for moderately disabled older adults with physical disabilities 16
Table A.7 Kano attributes of specialized home care services needs for severely disabled older adults with physical disabilities 17
Table A.8 Hierarchical model for prioritizing specialized home care service needs in fully self-care older adults with physical disabilities 18
Table A.9 Hierarchical model for prioritizing specialized home care service needs in mildly disability older adults with physical disabilities 19
Table A.10 Hierarchical model for prioritizing specialized home care service needs in moderate disability older adults with physical disabilities 20
Table A.11 Hierarchical model for prioritizing specialized home care service needs in severe disability older adults with physical disabilities 21

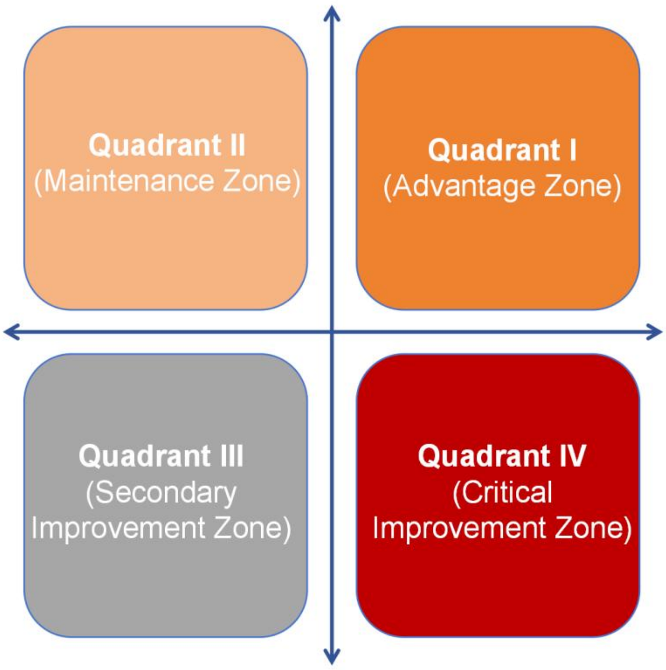

Figure A.1 Better-Worse matrix analysis model

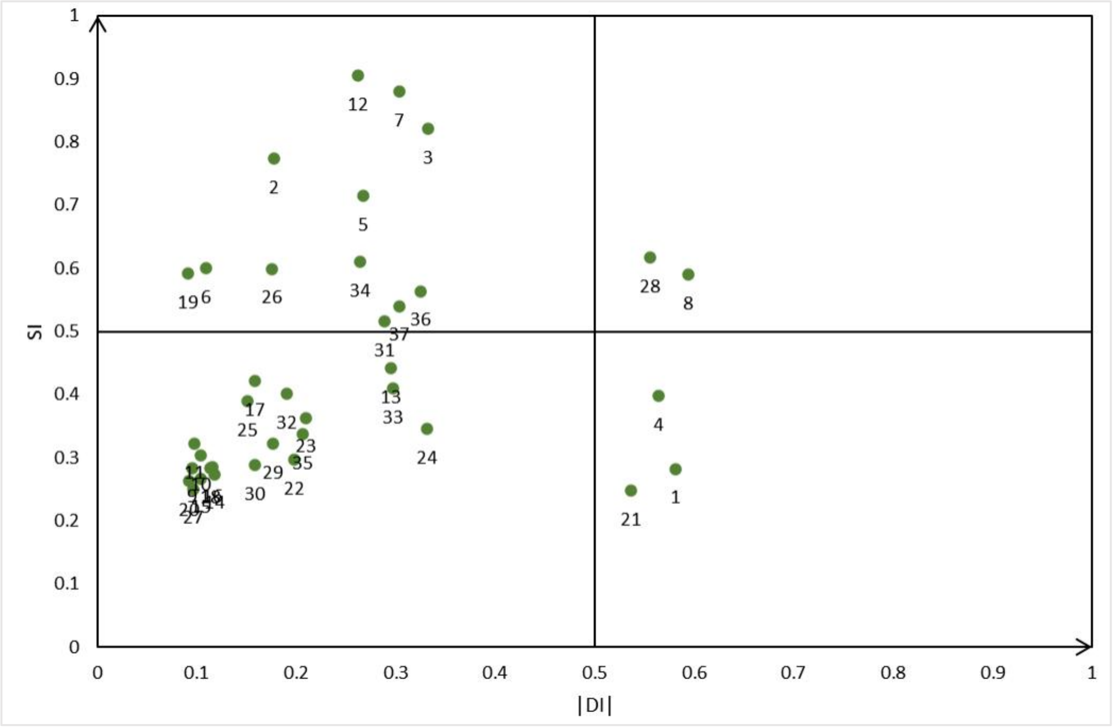

Figure A.2 Better-Worse matrix of specialized home care service needs for all older adults with physical disabilities

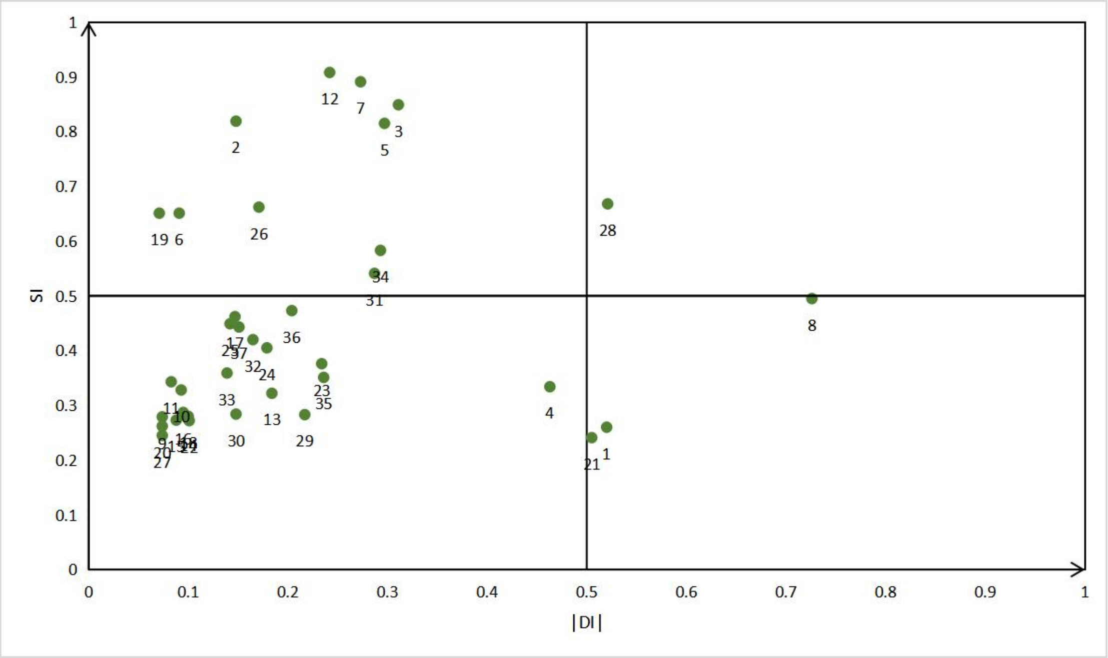

Figure A.3 Better-Worse matrix of specialized home care service needs for fully self-care older adults with physical disabilities

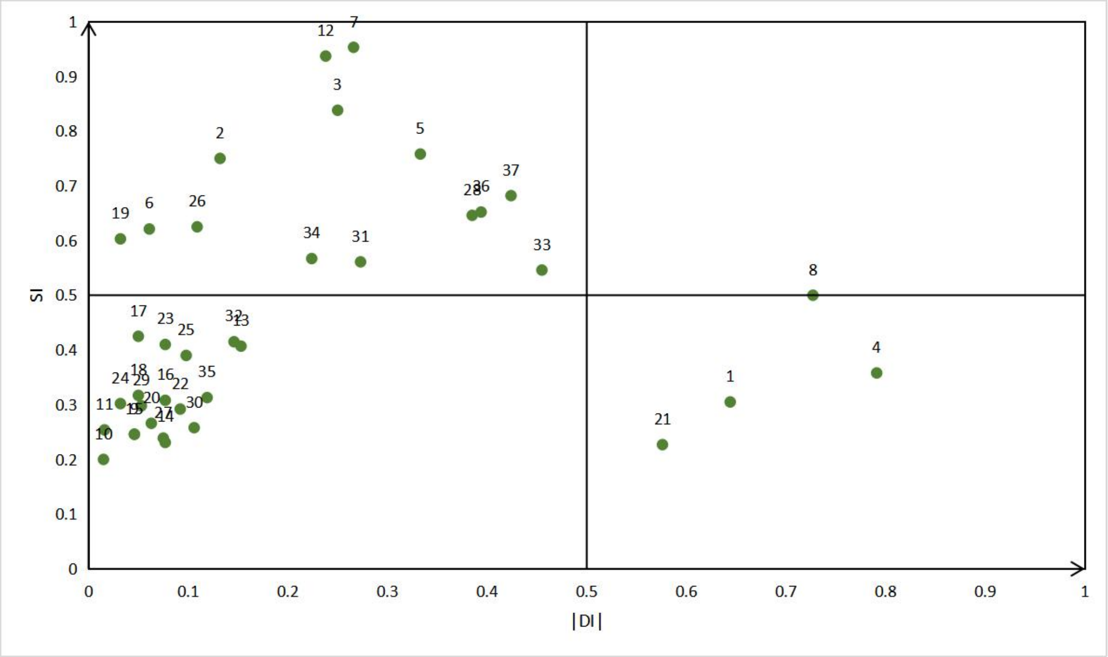

Figure A.4 Better-Worse matrix of specialized home care service needs for the mildly disabled older adults with physical disabilities

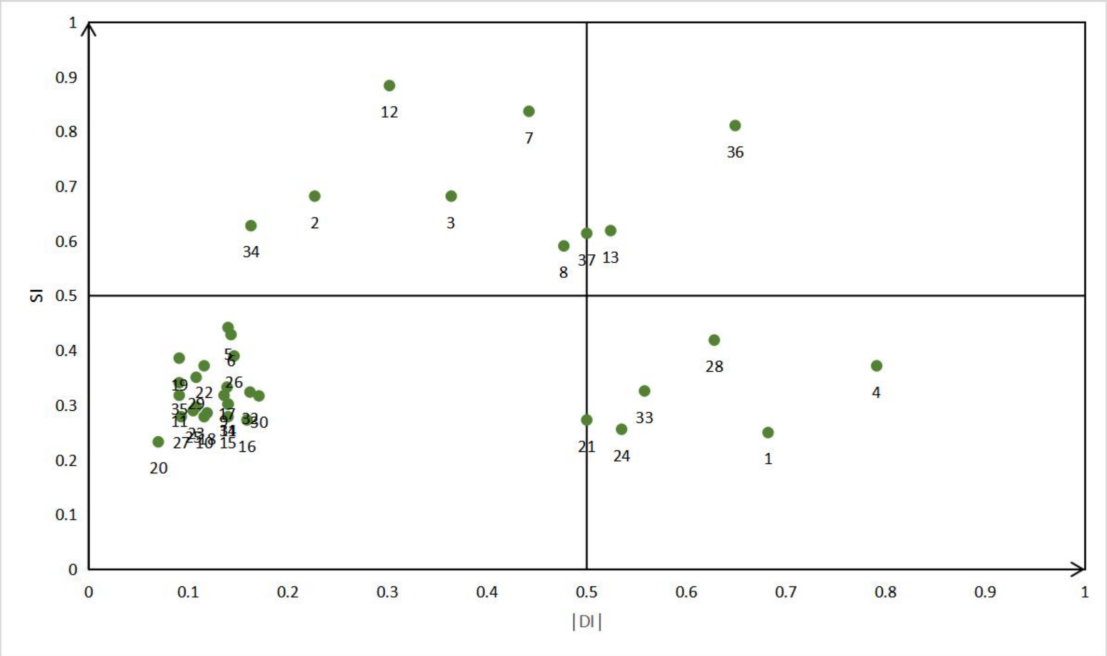

Figure A.5 Better-Worse matrix of specialized home care service needs for the moderately disabled older adults with physical disabilities

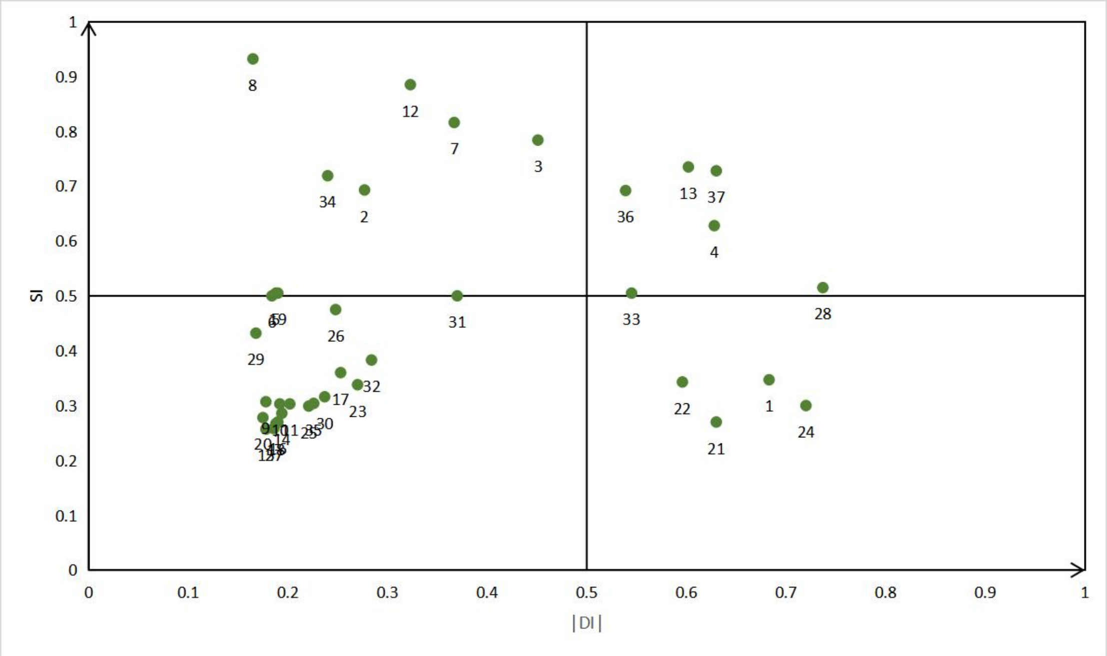

Figure A.6. Better-Worse matrix of specialized home care service needs for the severely disabled older adults with physical disabilities

Table A.1 Needs assessment questionnaire for specialized home care services

Do you need a professional caregiver to provide you with the following care services? Please rate your need on a 5-point scale of importance in the appropriate column (single-selected question).

	**Very satisfied**	**As it should be**	**It doesn't matter**	**Acceptable**	**Dissatisfied**
1. Item 1：Professionals come to your home to provide you with psychological care.					
How would you feel if a professional provided you with this service?	○	○	○	○	○
How would you feel if a professional did not provide you with this service?	○	○	○	○	○
2. Item 2：Professionals come to your home to provide you with nnursing knowledge and skills guidance.					
How would you feel if a professional provided you with this service?	○	○	○	○	○
How would you feel if a professional did not provide you with this service?	○	○	○	○	○
3. Item 3：Professionals come to your home to provide you with safety guidance.					
How would you feel if a professional provided you with this service?	○	○	○	○	○
How would you feel if a professional did not provide you with this service?	○	○	○	○	○
4. Item 4：Professionals come to your home to provide you with unctional rehabilitation training guidance.					
How would you feel if a professional provided you with this service?	○	○	○	○	○
How would you feel if a professional did not provide you with this service?	○	○	○	○	○
5. Item 5：Professionals come to your home to provide you with medication (medication safety) guidance.					
How would you feel if a professional provided you with this service?	○	○	○	○	○
How would you feel if a professional did not provide you with this service?	○	○	○	○	○

6. Item 6：Professionals come to your home to provide you with guidance on the use and selection of instruments.

How would you feel if a professional provided you with this service?	○	○	○	○	○
How would you feel if a professional did not provide you with this service?	○	○	○	○	○

7. Item 7：Professionals come to your home to provide you with disease prevention knowledge guidance.

How would you feel if a professional provided you with this service?	○	○	○	○	○
How would you feel if a professional did not provide you with this service?	○	○	○	○	○

8. Item 8：Professionals come to your home to provide you with health care knowledge guidance.

How would you feel if a professional provided you with this service?	○	○	○	○	○
How would you feel if a professional did not provide you with this service?	○	○	○	○	○

9. Item 9：Professionals come to your home to provide you with irst aid knowledge and skills guidance.

How would you feel if a professional provided you with this service?	○	○	○	○	○
How would you feel if a professional did not provide you with this service?	○	○	○	○	○

10. Item 10：Professionals come to your home to provide you with blood glucose measurement.

How would you feel if a professional provided you with this service?	○	○	○	○	○
How would you feel if a professional did not provide you with this service?	○	○	○	○	○

11. Item 11：Professionals come to your home to provide you with measurement of vital signs.

How would you feel if a professional provided you with this service?	○	○	○	○	○

How would you feel if a professional did not provide you with this service?	○	○	○	○	○

12. Item 12：Professionals come to your home to provide you with safety risk assessment for older adults.

How would you feel if a professional provided you with this service?	○	○	○	○	○
How would you feel if a professional did not provide you with this service?	○	○	○	○	○

13. Item 13：Professionals come to your home to provide you with oral care.

How would you feel if a professional provided you with this service?	○	○	○	○	○
How would you feel if a professional did not provide you with this service?	○	○	○	○	○

14. Item 14：Professionals come to your home to provide you with wound care.

How would you feel if a professional provided you with this service?	○	○	○	○	○
How would you feel if a professional did not provide you with this service?	○	○	○	○	○

15. Item 15：Professionals come to your home to provide you with injectables.

How would you feel if a professional provided you with this service?	○	○	○	○	○
How would you feel if a professional did not provide you with this service?	○	○	○	○	○

16. Item 16：Professionals come to your home to provide you with intravenous fluids.

How would you feel if a professional provided you with this service?	○	○	○	○	○
How would you feel if a professional did not provide you with this service?	○	○	○	○	○

17. Item 17：Professionals come to your home to provide you with blood specimen collection.

How would you feel if a professional provided you with this service?	○	○	○	○	○
How would you feel if a professional did not provide you with this service?	○	○	○	○	○

18. Item 18：Professionals come to your home to provide you with perineal care.

How would you feel if a professional provided you with this service?	○	○	○	○	○
How would you feel if a professional did not provide you with this service?	○	○	○	○	○

19. Item 19：Professionals come to your home to provide you with nutritional support nursing.

How would you feel if a professional provided you with this service?	○	○	○	○	○
How would you feel if a professional did not provide you with this service?	○	○	○	○	○

20. Item 20：Professionals come to your home to provide you with physical cooling.

How would you feel if a professional provided you with this service?	○	○	○	○	○
How would you feel if a professional did not provide you with this service?	○	○	○	○	○

21. Item 21：Professionals come to your home to provide you with sleep nursing.

How would you feel if a professional provided you with this service?	○	○	○	○	○
How would you feel if a professional did not provide you with this service?	○	○	○	○	○

22. Item 22：Professionals come to your home to provide you with catheterization nursing.

How would you feel if a professional provided you with this service?	○	○	○	○	○
How would you feel if a professional did not provide you with this service?	○	○	○	○	○

23. Item 23：Professionals come to your home to provide you with nasogastric care.

How would you feel if a professional provided you with this service?	○	○	○	○	○
How would you feel if a professional did not provide you with this service?	○	○	○	○	○

24. Item 24：Professionals come to your home to provide you with prevention and management of pressure injuries.

How would you feel if a professional provided you with this service?	○	○	○	○	○
How would you feel if a professional did not provide you with this service?	○	○	○	○	○

25. Item 25：Professionals come to your home to provide you with ostomy and fistula care

How would you feel if a professional provided you with this service?	○	○	○	○	○
How would you feel if a professional did not provide you with this service?	○	○	○	○	○

26. Item 26：Professionals come to your home to provide you with assisted defecation.

How would you feel if a professional provided you with this service?	○	○	○	○	○
How would you feel if a professional did not provide you with this service?	○	○	○	○	○

27. Item 27：Professionals come to your home to provide you with nebulized inhalation.

How would you feel if a professional provided you with this service?	○	○	○	○	○
How would you feel if a professional did not provide you with this service?	○	○	○	○	○

28. Item 28：Professionals come to your home to provide you with prevention and management of falls.

How would you feel if a professional provided you with this service?	○	○	○	○	○

How would you feel if a professional did not provide you with this service?	○	○	○	○	○

29. Item 29：Professionals come to your home to provide you with urination care.

How would you feel if a professional provided you with this service?	○	○	○	○	○
How would you feel if a professional did not provide you with this service?	○	○	○	○	○

30. Item 30：Professionals come to your home to provide you with putum discharge care.

How would you feel if a professional provided you with this service?	○	○	○	○	○
How would you feel if a professional did not provide you with this service?	○	○	○	○	○

31. Item 31：Professionals come to your home to provide you with oxygen therapy.

How would you feel if a professional provided you with this service?	○	○	○	○	○
How would you feel if a professional did not provide you with this service?	○	○	○	○	○

32. Item 32：Professionals come to your home to provide you with nursing care of indwelling tubes.

How would you feel if a professional provided you with this service?	○	○	○	○	○
How would you feel if a professional did not provide you with this service?	○	○	○	○	○

33. Item 33：Professionals come to your home to provide you with rehabilitation training.

How would you feel if a professional provided you with this service?	○	○	○	○	○
How would you feel if a professional did not provide you with this service?	○	○	○	○	○

34. Item 34：Professionals come to your home to provide you with acupoint massage.

How would you feel if a professional provided you with this service?	○	○	○	○	○
How would you feel if a professional did not provide you with this service?	○	○	○	○	○

35. Item 35：Professionals come to your home to provide you withapplication and cupping.

How would you feel if a professional provided you with this service?	○	○	○	○	○
How would you feel if a professional did not provide you with this service?	○	○	○	○	○

36. Item 36：Professionals come to your home to provide you with maintenance and care of living aids.

How would you feel if a professional provided you with this service?	○	○	○	○	○
How would you feel if a professional did not provide you with this service?	○	○	○	○	○

37. Item 37：Professionals come to your home to provide you with supervision of medical behavior.

How would you feel if a professional provided you with this service?	○	○	○	○	○
How would you feel if a professional did not provide you with this service?	○	○	○	○	○

Table A.2 Kano assessment framework

Function	Dysfunction				
	Very satisfied	As it should be	It doesn't matter	Acceptable	Dissatisfied
Very satisfied	Q	A	A	A	O
As it should be	R	I	I	I	M
It doesn't matter	R	I	I	I	M
Acceptable	R	I	I	I	M
Dissatisfied	R	R	R	R	Q

Note: M - Must-be Needs, O - One-dimensional Needs, A - Attractive Needs, I - Indifferent Needs, R-Reverse Needs, Q - Question Quality

Table A.3 Kano attributes of specialized home care services needs for older adults with physical disabilities

Items		A	M	O	R	Q	I	KANO attributes
1.	Psychological care	85	237	58	22	4	128	M
2.	Nursing knowledge and skills guidance	335	23	70	4	7	95	A
3.	Safety guidance	279	22	153	3	5	72	A
4.	Functional rehabilitation training guidance	71	158	137	7	4	157	M
5.	Medication (medication safety) guidance	309	79	58	16	5	67	A
6.	Guidance on the use and selection of instruments	264	11	45	13	6	195	A
7.	Disease prevention knowledge guidance	336	39	118	6	12	23	A
8.	Health care knowledge guidance	210	212	97	4	10	1	M
9.	First aid knowledge and skills guidance	114	16	34	6	6	358	I
10.	Blood glucose measurement	117	16	37	16	9	339	I
11.	Measurement of vital signs	127	13	37	19	6	332	I
12.	Safety risk assessment for older adults	334	15	115	6	32	32	A
13.	Oral care	120	49	93	32	20	220	I
14.	Wound care	100	20	41	13	6	354	I
15.	Injectables	99	15	39	11	6	364	I
16.	Intravenous fluids	109	22	38	12	6	347	I
17.	Blood specimen collection	104	16	37	193	6	178	R
18.	Perineal care	102	19	37	33	9	334	I
19.	Nutritional support nursing	254	12	32	2	49	185	A
20.	Physical cooling	103	15	32	11	10	363	I
21.	Sleep nursing	92	243	37	5	8	149	M
22.	Catheterization nursing	107	57	43	23	6	298	I
23.	Nasogastric care	85	32	41	171	16	189	I
24.	Prevention and management of pressure injuries	94	88	43	125	13	171	I
25.	Ostomy and fistula care	92	13	37	195	7	190	R
26.	Assisted defecation	230	14	76	15	8	191	A
27.	Nebulized inhalation	95	16	34	8	9	372	I
28.	Prevention and management of falls	101	70	216	4	16	127	O
29.	Urination care	110	45	34	67	20	258	I
30.	Sputum discharge care	102	38	40	36	5	313	I
31.	Oxygen therapy	130	11	140	6	5	242	I
32.	Nursing care of indwelling tubes	93	20	46	182	5	188	I
33.	Rehabilitation training	138	81	69	24	5	217	I
34.	Acupoint massage	228	48	89	10	4	155	A
35.	Application and cupping	125	57	52	4	4	292	I
36.	Maintenance and care of living aids	127	18	130	42	36	181	I
37.	Supervision of medical behavior	140	20	134	22	5	213	I

Note: M - Must-be Needs, O - One-dimensional Needs, A - Attractive Needs, I - Indifferent Needs, R-Reverse Needs, Q - Question Quality

Table A.4 Kano attributes of specialized home care services needs for fully self-care older adults with physical disabilities

Items		A	M	O	R	Q	I	KANO attributes
1.	Psychological care	53	132	26	13	1	93	M
2.	Nursing knowledge and skills guidance	217	9	37	4	4	47	A
3.	Safety guidance	180	12	85	3	3	35	A
4.	Functional rehabilitation training guidance	23	63	81	5	2	144	I
5.	Medication (medication safety) guidance	212	55	35	13	2	1	A
6.	Guidance on the use and selection of instruments	175	2	26	7	2	106	A
7.	Disease prevention knowledge guidance	211	19	66	3	4	15	A
8.	Health care knowledge guidance	83	154	69	4	7	1	M
9.	First aid knowledge and skills guidance	69	5	18	4	2	220	I
10.	Blood glucose measurement	76	5	23	10	6	198	I
11.	Measurement of vital signs	82	3	22	13	2	196	I
12.	Safety risk assessment for older adults	204	8	63	2	22	19	A
13.	Oral care	64	25	27	31	4	167	I
14.	Wound care	63	9	22	7	2	215	I
15.	Injectables	62	5	22	8	2	219	I
16.	Intravenous fluids	67	8	21	8	3	211	I
17.	Blood specimen collection	64	6	21	132	2	93	R
18.	Perineal care	60	8	21	26	3	200	I
19.	Nutritional support nursing	165	2	18	2	35	96	A
20.	Physical cooling	62	4	19	6	3	224	I
21.	Sleep nursing	52	134	23	4	3	102	M
22.	Catheterization nursing	60	9	21	17	3	208	I
23.	Nasogastric care	53	25	21	109	12	98	R
24.	Prevention and management of pressure injuries	54	11	23	118	10	102	R
25.	Ostomy and fistula care	56	2	23	139	3	95	R
26.	Assisted defecation	154	4	48	9	4	99	A
27.	Nebulized inhalation	57	4	19	3	5	230	I
28.	Prevention and management of falls	56	11	149	2	9	91	O
29.	Urination care	51	34	22	56	4	151	I
30.	Sputum discharge care	63	21	25	6	2	201	I
31.	Oxygen therapy	82	2	88	3	1	142	I
32.	Nursing care of indwelling tubes	55	7	24	128	2	102	R
33.	Rehabilitation training	75	10	31	21	2	179	I
34.	Acupoint massage	123	32	60	3	1	99	A
35.	Application and cupping	75	39	35	3	2	164	I
36.	Maintenance and care of living aids	83	9	47	39	4	136	I
37.	Supervision of medical behavior	99	10	36	11	2	160	I

Note: M - Must-be Needs, O - One-dimensional Needs, A - Attractive Needs, I - Indifferent Needs, R-Reverse Needs, Q - Question Quality

Table A.5 Kano attributes of specialized home care services needs for mildly disabled older adults with physical disabilities

Items	A	M	O	R	Q	I	KANO attributes
1. Psychological care	**12**	**32**	**6**	**8**	**1**	**9**	**M**
2. Nursing knowledge and skills guidance	**45**	**3**	**6**	**0**	**0**	**14**	**A**
3. Safety guidance	**41**	**1**	**16**	**0**	**0**	**10**	**A**
4. Functional rehabilitation training guidance	**12**	**41**	**12**	**1**	**0**	**2**	**M**
5. Medication (medication safety) guidance	**44**	**16**	**6**	**1**	**1**	**0**	**A**
6. Guidance on the use and selection of instruments	37	0	4	1	1	25	A
7. Disease prevention knowledge guidance	45	1	16	2	2	2	A
8. Health care knowledge guidance	18	33	15	0	2	0	M
9. First aid knowledge and skills guidance	14	1	2	1	2	48	I
10. Blood glucose measurement	12	0	1	3	0	52	I
11. Measurement of vital signs	15	0	1	4	1	47	I
12. Safety risk assessment for older adults	45	1	14	1	4	3	A
13. Oral care	21	6	3	1	8	29	I
14. Wound care	11	1	4	2	1	49	I
15. Injectables	13	0	3	2	1	49	I
16. Intravenous fluids	17	2	3	2	1	43	I
17. Blood specimen collection	15	0	2	26	2	23	R
18. Perineal care	16	0	3	5	3	41	I
19. Nutritional support nursing	36	0	2	0	5	25	A
20. Physical cooling	14	1	3	1	3	46	I
21. Sleep nursing	12	35	3	1	1	16	M
22. Catheterization nursing	14	1	5	2	1	45	I
23. Nasogastric care	13	0	3	28	1	23	R
24. Prevention and management of pressure injuries	17	0	2	4	1	44	I
25. Ostomy and fistula care	13	1	3	25	2	24	R
26. Assisted defecation	33	0	7	3	1	24	A
27. Nebulized inhalation	13	2	3	0	1	49	I
28. Prevention and management of falls	17	0	25	0	3	23	O
29. Urination care	15	1	2	9	2	39	I
30. Sputum discharge care	14	4	3	1	1	45	I
31. Oxygen therapy	19	0	18	1	1	29	I
32. Nursing care of indwelling tubes	13	2	4	26	1	22	R
33. Rehabilitation training	22	16	14	1	1	14	A
34. Acupoint massage	27	4	11	0	1	25	A
35. Application and cupping	19	6	2	1	0	40	I
36. Maintenance and care of living aids	17	0	26	2	0	23	O
37. Supervision of medical behavior	18	1	27	1	1	20	I

Note: M - Must-be Needs, O - One-dimensional Needs, A - Attractive Needs, I - Indifferent Needs, R-Reverse Needs, Q - Question Quality

Table A.6 Kano attributes of specialized home care services needs for moderately disabled older adults with physical disabilities

Items	A	M	O	R	Q	I	KANO attributes
1. Psychological care	5	24	6	0	1	9	M
2. Nursing knowledge and skills guidance	22	2	8	0	1	12	A
3. Safety guidance	16	2	14	0	1	12	A
4. Functional rehabilitation training guidance	7	25	9	1	1	2	M
5. Medication (medication safety) guidance	15	2	4	1	1	22	I
6. Guidance on the use and selection of instruments	14	2	4	2	1	22	I
7. Disease prevention knowledge guidance	22	5	14	0	2	2	A
8. Health care knowledge guidance	23	18	3	0	1	0	A
9. First aid knowledge and skills guidance	10	2	4	0	1	28	I
10. Blood glucose measurement	10	3	2	1	1	28	I
11. Measurement of vital signs	12	2	2	0	1	28	I
12. Safety risk assessment for older adults	26	1	12	2	0	4	A
13. Oral care	14	10	12	0	3	6	A
14. Wound care	10	3	3	1	1	27	I
15. Injectables	8	2	4	1	1	29	I
16. Intravenous fluids	8	3	4	0	1	29	I
17. Blood specimen collection	9	2	3	8	1	22	I
18. Perineal care	10	3	2	1	2	27	I
19. Nutritional support nursing	16	3	1	0	1	24	I
20. Physical cooling	9	2	1	1	1	31	I
21. Sleep nursing	11	21	1	0	1	11	M
22. Catheterization nursing	14	3	2	1	1	24	I
23. Nasogastric care	8	1	3	7	1	25	I
24. Prevention and management of pressure injuries	9	21	2	1	1	11	M
25. Ostomy and fistula care	10	3	1	6	1	24	I
26. Assisted defecation	13	3	3	2	2	22	I
27. Nebulized inhalation	11	3	1	1	1	28	I
28. Prevention and management of falls	11	20	7	1	1	5	M
29. Urination care	12	3	1	1	7	21	I
30. Sputum discharge care	11	5	2	3	1	23	I
31. Oxygen therapy	9	2	4	1	1	28	I
32. Nursing care of indwelling tubes	9	3	3	7	1	22	I
33. Rehabilitation training	10	20	4	1	1	9	M
34. Acupoint massage	25	5	2	1	1	11	A
35. Application and cupping	13	2	2	0	1	27	I
36. Maintenance and care of living aids	8	2	22	0	8	5	O
37. Supervision of medical behavior	7	2	20	0	1	15	O

Note: M - Must-be Needs, O - One-dimensional Needs, A - Attractive Needs, I - Indifferent Needs, R-Reverse Needs, Q - Question Quality

Table A.7 Kano attributes of specialized home care services needs for severely disabled older adults with physical disabilities

Items	A	M	O	R	Q	I	KANO attributes
1. Psychological care	15	49	20	1	1	17	M
2. Nursing knowledge and skills guidance	51	9	19	0	2	22	A
3. Safety guidance	41	7	39	0	1	15	A
4. Functional rehabilitation training guidance	29	29	35	0	1	9	O
5. Medication (medication safety) guidance	38	6	13	1	1	44	I
6. Guidance on the use and selection of instruments	38	7	11	3	2	42	I
7. Disease prevention knowledge guidance	58	14	22	1	4	4	A
8. Health care knowledge guidance	86	7	10	0	0	0	A
9. First aid knowledge and skills guidance	21	8	10	1	1	62	I
10. Blood glucose measurement	19	8	11	2	2	61	I
11. Measurement of vital signs	18	8	12	2	2	61	I
12. Safety risk assessment for older adults	59	5	26	1	6	6	A
13. Oral care	21	8	51	0	5	18	O
14. Wound care	16	7	12	3	2	63	I
15. Injectables	16	8	10	0	2	67	I
16. Intravenous fluids	17	9	10	2	1	64	I
17. Blood specimen collection	16	8	11	27	1	40	I
18. Perineal care	16	8	11	1	1	66	I
19. Nutritional support nursing	37	7	11	0	8	40	I
20. Physical cooling	18	8	9	3	3	62	I
21. Sleep nursing	17	53	10	0	3	20	M
22. Catheterization nursing	19	44	15	3	1	21	M
23. Nasogastric care	11	6	14	27	2	43	I
24. Prevention and management of pressure injuries	14	56	16	2	1	14	M
25. Ostomy and fistula care	13	7	10	25	1	47	I
26. Assisted defecation	30	7	18	1	1	46	I
27. Nebulized inhalation	14	7	11	4	2	65	I
28. Prevention and management of falls	17	39	34	1	3	9	M
29. Urination care	32	7	9	1	7	47	I
30. Sputum discharge care	14	8	10	26	1	44	I
31. Oxygen therapy	20	7	30	1	2	43	I
32. Nursing care of indwelling tubes	16	8	15	21	1	42	I
33. Rehabilitation training	31	35	20	1	1	15	M
34. Acupoint massage	53	7	16	6	1	20	A
35. Application and cupping	18	10	13	0	1	61	I
36. Maintenance and care of living aids	19	7	35	1	24	17	O
37. Supervision of medical behavior	16	7	51	10	1	18	O

Note: M - Must-be Needs, O - One-dimensional Needs, A - Attractive Needs, I - Indifferent Needs, R-Reverse Needs, Q - Question Quality

Table A.8 Hierarchical model for prioritizing specialized home care service needs in fully self-care older adults with physical disabilities

Quadrant	Items	Priority ranking
	Item 8: health care knowledge guidance	1
**Critical improvement zone**	Item 1: psychological care	2
	Item 21: sleep nursing	3
**Advantage zone**	Item 28: prevention and management of falls	4
	Item 12: safety risk assessment for older adults	5
	Item 7: disease prevention knowledge guidance	6
	Item 3: safety guidance	7
	Item 2: nursing knowledge and skills guidance	8
	Item 5: medication (medication safety) guidance	9
**Maintenance zone**	Item 26: assisted defecation	10
	Item 6: guidance on the use and selection of instruments	11
	Item 19: nutritional support nursing	12
	Item 34: acupoint massage	13
	Item 31: oxygen therapy	14

Table A.9 Hierarchical model for prioritizing specialized home care service needs in mildly disability older adults with physical disabilities

Quadrant	Items	Priority ranking
**Critical improvement zone**	Item 4: functional rehabilitation training guidance	1
	Item 1: psychological care	2
	Item 21: sleep nursing	3
**Advantage zone**	Item 8: health care knowledge guidance	4
	Item 7: disease prevention knowledge guidance	5
	Item 12: safety risk assessment for older adults	6
	Item 3: safety guidance	7
	Item 5: medication (medication safety) guidance	8
	Item 2: nursing knowledge and skills guidance	9
	Item 37: supervision of medical behavior	10
**Maintenance zone**	Item 36: maintenance and care of living aids	11
	Item 28: prevention and management of falls	12
	Item 26: assisted defecation	13
	Item 6: guidance on the use and selection of instruments	14
	Item 19: nutritional support nursing	15
	Item 34: acupoint massage	16
	Item 31: oxygen therapy	17
	Item 33: rehabilitation training	18

Table A.10 Hierarchical model for prioritizing specialized home care service needs in moderate disability older adults with physical disabilities

Quadrant	Items	Priority ranking
**Critical improvement zone**	Item 28：prevention and management of falls	1
	Item 4：functional rehabilitation training guidance	2
	Item 33：rehabilitation training	3
	Item 21: sleep nursing	4
	Item 24: prevention and management of pressure injuries	5
**Advantage zone**	Item 1: psychological care	6
	Item 36: maintenance and care of living aids	7
	Item 13: oral care	8
	Item 37: supervision of medical behavior	9
**Maintenance zone**	Item 12: safety risk assessment for older adults	10
	Item 7: disease prevention knowledge guidance	11
	Item 2: nursing knowledge and skills guidance	12
	Item 3: safety guidance	13
	Item 34: acupoint massage	14
	Item 8: health care knowledge guidance	15

Table A.11 Hierarchical model for prioritizing specialized home care service needs in severe disability older adults with physical disabilities

Quadrant	Items	Priority ranking
Critical improvement zone	Item 1： psychological care	1
	Item 22: catheterization nursing	2
	Item 24: prevention and management of pressure injuries	3
	Item 21: sleep nursing	4
	Item 13: oral care	5
Advantage zone	Item 37: supervision of medical behavior	6
	Item 36: maintenance and care of living aids	7
	Item 4: functional rehabilitation training guidance	8
	Item 28: prevention and management of falls	9
	Item 33: rehabilitation training	10
Maintenance zone	Item 8: health care knowledge guidance	11
	Item 12: safety risk assessment for older adults	12
	Item 7: disease prevention knowledge guidance	13
	Item 3: safety guidance	14
	Item 34: acupoint massage	15
	Item 2: nursing knowledge and skills guidance	16
	Item 5: medication (medication safety) guidance	17
	Item 19: nutritional support nursing	18
